# Efficacy of Whole-Body Electromyostimulation (WB-EMS) on Body Composition and Muscle Strength in Non-athletic Adults. A Systematic Review and Meta-Analysis

**DOI:** 10.3389/fphys.2021.640657

**Published:** 2021-02-26

**Authors:** Wolfgang Kemmler, Mahdieh Shojaa, James Steele, Joshua Berger, Michael Fröhlich, Daniel Schoene, Simon von Stengel, Heinz Kleinöder, Matthias Kohl

**Affiliations:** ^1^Institute of Medical Physics, University of Erlangen-Nürnberg, Erlangen, Germany; ^2^Ukactive Research Institute, London, United Kingdom; ^3^Faculty of Sport, Health, and Social Sciences, Solent University, Southampton, United Kingdom; ^4^Department of Sports Science, Technische Universität Kaiserslauter, Kaiserslautern, Germany; ^5^German Sport University Cologne, Cologne, Germany; ^6^Faculty Medical and Life Sciences, University of Furtwangen, Villingen-Schwenningen, Germany

**Keywords:** whole-body electromyostimulation, exercise, body composition, lean body mass, body fat mass, muscle strength

## Abstract

This systematic review and meta-analysis set out to determine the efficacy on whole-body electromyostimulation (WB-EMS) on body composition and strength parameters in non-athletic cohorts. A systematic review of the literature according to the PRISMA statement included (a) controlled trials, (b) WB-EMS trials with at least one exercise and one control group, (c) WB-EMS as primary physical intervention, (d) WB-EMS with at least six electrodes covering most muscle groups, (e) non-athletic cohorts. We searched eight electronic databases up to June 30, 2020, without language restrictions. Standardized mean differences (SMD) for muscle mass parameters, total body fat mass, maximum leg extension, and trunk extension strength were defined as outcome measures. In summary, 16 studies with 19 individual WB-EMS groups representing 897 participants were included. Studies vary considerably with respect to age, BMI, and physical conditions. Impulse protocols of the studies were roughly comparable, but training frequency (1–5 sessions/week) and intervention length (6–54 weeks) differed between the studies. SMD average was 1.23 (95%-CI: 0.71–1.76) for muscle mass, 0.98 (0.74–1.22) for maximum leg, and 1.08 (0.78–1.39) for maximum trunk extension strength changes (all *p* < 0.001). SMD for body fat changes (−0.40, [−0.98 to 0.17]), however, did not reach significance. *I*^2^ and Q-statistics revealed substantial heterogeneity of muscle and fat mass changes between the trials. However, rank and regression tests did not indicate positive evidence for small-study bias and funnel plot asymmetries. This work provided further evidence for significant, large-sized effects of WB-EMS on muscle mass and strength parameters, but not on body fat mass.

**Clinical Trial Registration:** ClinicalTrials.gov, PROSPERO; ID: CRD42020183059.

## Introduction

Whole-body electromyostimulation (WB-EMS) is an ever more popular training technology that can stimulate multiple muscle groups simultaneously with regionally dedicated intensity. Although there are a multitude of possible protocols, WB-EMS in its most common setting applies short impulse phases (4–6 s) intermitted by short phases of rest (4 s) with moderate to high impulse intensity for about 20 min. However, while most protocols use similar impulse settings (e.g., bipolar, 80–85 Hz, 300–400 μs, intermitted), two fundamentally different WB-EMS concepts have evolved and should be considered when classifying WB-EMS. One strategy predominately used in athletic performance (e.g., Filipovic et al., 2015, 2016, 2019; Wirtz et al., 2016, 2019; Amaro-Gahete et al., 2018a,b; Micke et al., 2018; Ludwig et al., 2020), but rarely applied in the health and fitness domain (Amaro-Gahete et al., 2019a; Pano-Rodriguez et al., 2020a,b), combined different stimulation parameters (i.e., frequencies, pulse width, and current cycles) and prescribes high voluntary loads superimposed by WB-EMS with an impulse intensity that just allows the proper application of the target exercise (e.g., weighted squats, jumps). In diametric contrast, the more popular WB-EMS strategy, almost exclusively applied by commercial WB-EMS suppliers, focuses on negligible to low^1^ effort voluntary workload by gentle movements and (adjuvant) moderate-high impulse intensities, i.e., “electric current” not voluntary workload providing the dominant effect. However, independently of this aspect, WB-EMS can be classified predominately as a resistance type exercise. Correspondingly, most studies determined the effect of WB-EMS on lean body mass (LBM), muscle strength, and function (e.g., Kemmler et al., 2014, 2016b; Amaro-Gahete et al., 2019a; Jee, 2019; Pano-Rodriguez et al., 2020a), but also on body fat (e.g., Vatter, 2010; Kemmler et al., 2018a; Schink et al., 2018; Jee, 2019; Bellia et al., 2020; Ricci et al., 2020) and less frequently, albeit largely successfully, to address cardiometabolic parameters (e.g., Kemmler et al., 2016c,d; Jee, 2018; Bellia et al., 2020) and metabolism (e.g., Kemmler et al., 2010b, 2012). In parallel, although several studies focus on athletic performance in younger adults (e.g., Filipovic et al., 2016, 2019; Amaro-Gahete et al., 2018b; D'Ottavio et al., 2019; Wirtz et al., 2019; Ludwig et al., 2020), the vast majority of WB-EMS trials address the health and fitness domain in predominately untrained, middle-aged to older adults (Kemmler et al., 2020b). This core client group of commercial WB-EMS providers (EMS-Training.de, 2017) might be predominately attracted by the perceived time efficiency, low mechanical demands, joint “friendliness,”^2^ and individual scalability (Kemmler et al., 2020b) of this training technology. However, the decisive aspect is still the efficacy of the training technology on its core outcomes. To date, the considerable amount of randomized or non-randomized controlled WB-EMS trials addressing body composition, muscle strength, and function has reported promising results (e.g., Kemmler et al., 2014; Schink et al., 2018; Weissenfels et al., 2018; Jee, 2019; Ludwig et al., 2019; Willert et al., 2019; Bellia et al., 2020; Ricci et al., 2020). However, in order to generate decisive evidence, a meta-analysis was seen as the most adequate study type (Kemmler et al., 2020a). Due to its high degree of standardization^3^ and the corresponding homogeneity with respect to the impulse protocols, WB-EMS (see above) might be a perfect candidate for a meta-analysis in the otherwise critical (qua heterogeneous) area of sports and exercise (Kemmler, 2013; Gentil et al., 2017). The aim of the present study was thus to provide further evidence for the effectiveness of WB-EMS to impact body composition, muscle strength, and function.

Our primary hypothesis was that WB-EMS generates a positive, statistically significant effect on lean body mass or related parameters. Our secondary hypotheses were that WB-EMS generates a positive effect on (a) total body fat mass, (b) maximum leg extension strength, or (c) trunk extension strength.

## Methods

### Literature Search and Study Selection

This review followed the guidelines recommended by the Preferred Reporting Items for Systematic Reviews and Meta-Analyses (PRISMA) statement (Moher et al., 2015), and assessing the methodological quality of systematic reviews checklist (AMSTAR-2) (Shea et al., 2017). It was registered in advance in the international prospective register of systematic reviews (PROSPERO; ID: CRD42020183059). Literature searches with no language restriction were conducted through PubMed, Scopus, Web of Science, Cochrane, Science Direct, Eric, ProQuest, and Primo for all articles published up to June 30, 2020. The search strategy utilized the intervention and outcome approach. The literature search was constructed around search terms for “Whole-Body Electromyostimulation,” “muscle strength,” and “body composition.”

A standard protocol for this search was developed and controlled vocabulary (MESH term for MEDLINE) was used. We used key words and their synonyms to sensitize the search by applying the following query: (“WB-EMS” or “Whole-Body Electromyostimulation” or “electromyostimulation” or “electrical muscle stimulation” or “electro-myo-stimulation” or “integral electrical stimulation” or “whole-body electrical muscle stimulation” or “electric muscle stimulation therapy”) AND (“body composition” or “body fat distribution” or “obesity” or “fat mass” or “body mass index” or “muscle mass” or “sarcopenia” or “muscular atrophy”) AND (“physical fitness” or “physical” or “fitness” or “muscle strength” or “muscle Inhibition” or “arthrogenic muscle inhibition” or “functional ability” or “daily living activity”) AND (“clinical trial” or “randomized clinical trial”).

Furthermore, reference lists of the included articles were searched manually to locate additional relevant studies. Unpublished reports or articles for which only abstracts were available were not considered. Duplicate publications from single trials were identified by comparing author names, intervention comparisons, publication dates, sample sizes, and outcomes. Authors of trials that were potentially eligible were contacted by e-mail for any missing data (e.g., mean change of BMD or SD) or for clarification of the study design, intervention, or study outcomes. Five out of seven authors responded to our queries (Vatter, 2010; Schink et al., 2018; Ludwig et al., 2019; Bellia et al., 2020; Ricci et al., 2020).

### Inclusion and Exclusion Criteria

Articles meeting the following criteria were included: (1) randomized or non-randomized controlled trials; (2) at least one group with WB-EMS intervention superimposed on low effort voluntary loads^4^ only vs. inactive, sham, or placebo control group; (3) WB-EMS applied to at least for six major muscle groups; (4) body composition and/or muscle strength as outcomes measurements; (5) WB-EMS as the primary physical intervention.

Exclusion criteria were: (1) athletic participants; (2) WB-EMS that did not cover upper trunk; (3) duplicate information or preliminary data from a subsequently published study; (4) review articles, case reports, editorials, conference abstracts, and letters; (5) WB-EMS application superimposed on otherwise high effort voluntary loads.

### Data Extraction

Titles and abstracts were screened by an independent reviewer (MS) to exclude irrelevant studies. Two reviewers (WK & MS) separately and independently evaluated full-text articles and extracted data from the included studies. Disagreement was resolved by discussion between the two reviewers; if they could not reach a consensus, a third reviewer was consulted (SvS). The following data were extracted: publication details (i.e., the first author's name, title, country and publication year), details of the study (e.g., design, objectives, sample size for each group), description of intervention (e.g., intervention period, frequency, duration), compliance (including number of withdrawals), adverse effects, risk assessment, body composition, and muscle strength values at baseline and study completion.

### Outcomes

Outcome measures were lean body mass/muscle mass, total body fat mass, maximum leg extension, and maximum trunk extension strength without limitations on the methods of measurement.

### Quality Assessment

The methodological quality of the studies included was evaluated by five reviewers (WK, JS, MS, MF, JB) using the Physiotherapy Evidence Database (PEDro) scale risk of bias tool (Sherrington et al., 2000; de Morton, 2009) and the Tool for the assEssment of Study qualiTy and reporting in EXercise (TESTEX) (Smart et al., 2015). The latter scale applies 12 criteria, some of which have more than one possible point, for a maximum score of 15 points. The PEDro scale is composed of 11 items of which only 10 items are scored (0/1). Both scales refer to randomization, allocation concealment, similarity at baseline, blinding of participants, staff and assessors, incomplete outcome data, intention-to-treat analysis, between-group comparison, and measure of variability. However, the TESTEX scale has some extra points regarding the exercise intervention characteristics (i.e., intensity, duration, frequency), and activity monitoring in control groups that were deemed appropriate given the intervention examined (i.e., WB-EMS). Discrepancies were discussed with a review author (SvS) until a consensus was reached. Of importance, articles by our group (WK, DS, MS, SvS) were consistently evaluated by other working groups (MF, JB, and/or JS).

### Data Synthesis

If the confidence interval (CI) was reported, it was converted to the standard deviation (SD) using the standardized formula (Higgins and Green, 2011). In case of data unavailability, the exact *P*-value of the absolute change of desired outcomes was obtained by computing the SD according to the change. In the case of unreported P-value, the SDs were calculated using the following equation: √[SD^2^_pre_+SD^2^_post_-(2× corr× SD_pre_ × SD_post_)]. The correlation coefficient corr is computed from studies with complete data or with all SDs specified (Higgins and Green, 2011). SD_pre_ and SD_post_ are the baseline and final standard deviation, respectively. If the absolute mean change of outcomes was unavailable, this was calculated using the difference between pre- and post-intervention scores.

### Statistical Analysis

All analyses were conducted using the metafor package (Viechtbauer, 2010) of the statistical software R (version 4.0.3; R Core Team, 2020). Random effect models were applied and standardized mean differences (SMDs) were calculated as effect size along with 95% CIs (Viechtbauer, 2010). Statistical heterogeneity was assessed using *Q* and *I*^2^ statistics (low: 0–39%, moderate: 40–59%, substantial: >60% [Higgins and Green, 2011]). We divided the control group into smaller groups in cases where there was more than one intervention group (Jee, 2019; Ludwig et al., 2019). To explore potential small-study biases and asymmetry, we inspected funnel plots. *P*-values < 0.05 were considered statistically significant. All data are presented as mean value (MV) ± standard deviation (SD) or MV and 95% confidence interval (95% CI). Since there were different values of the correlation coefficient, a sensitivity analysis was performed (minimum, mean, or maximum) to assess whether the overall result of the analysis was robust to the use of imputed standard deviations.

## Results

**Figure 1:**
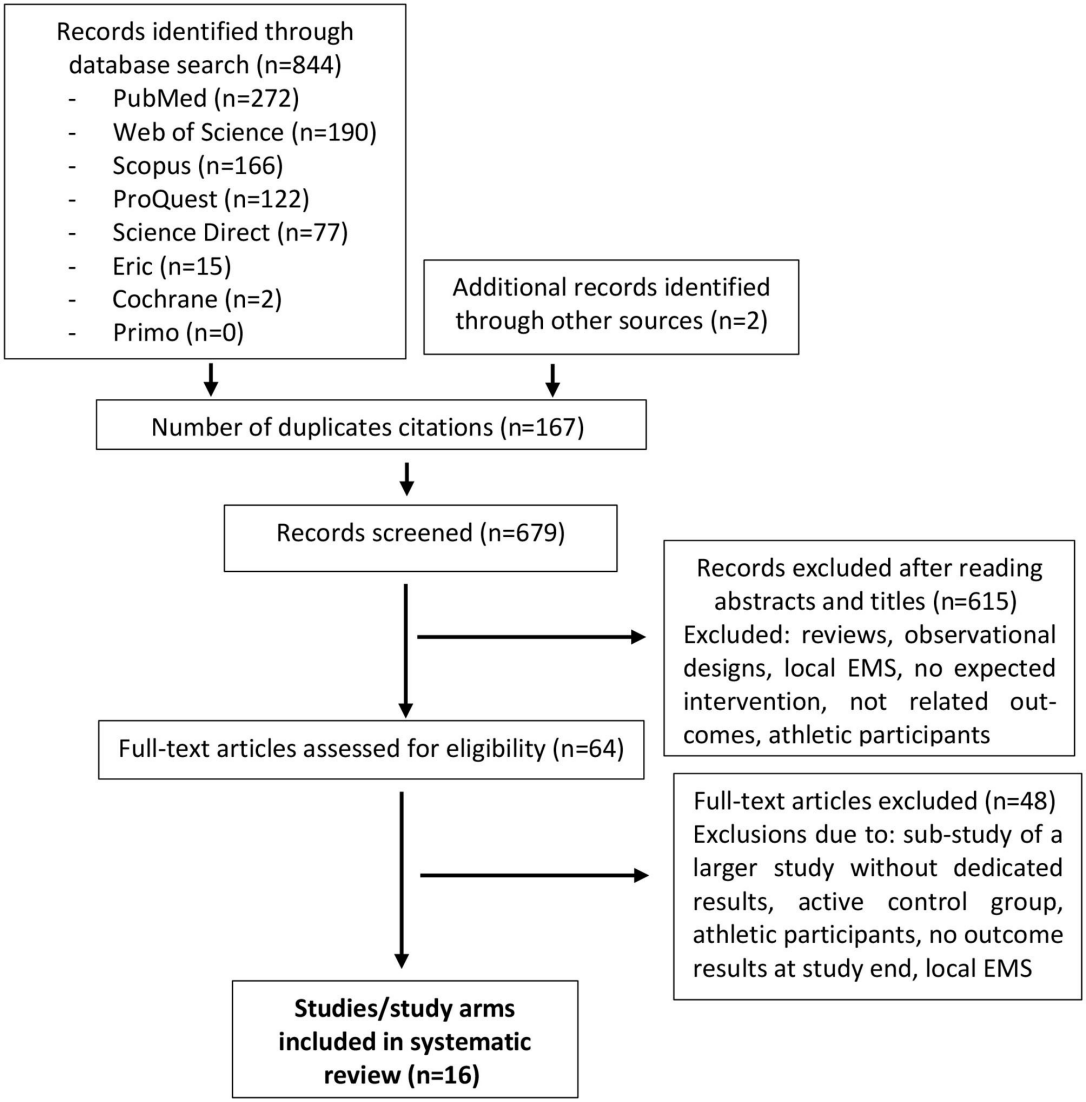
Flow diagram of search process.

Figure 1 presents the flow chart of study selection based on the PRISMA statement (Moher et al., 2009). The initial search identified 844 publications. The full text of 64 potentially relevant articles was then checked, with 16 articles then being included in this systematic review. Two included studies contained English abstracts, but with German (Kemmler et al., 2010a, 2017b) full texts.

### Study and Participant Characteristics

Full descriptive details of the studies included are shown in Table 2. Sixteen studies were included in this systematic review and meta-analysis, comprising 19 individual training groups based on our eligibility criteria (Kemmler et al., 2010a,b, 2014, 2016c,d, 2018a,b; Vatter, 2010; Kemmler and von Stengel, 2013; Schink et al., 2018; Weissenfels et al., 2018; Jee, 2019; Bellia et al., 2020; Kim and Jee, 2020; Ludwig et al., 2020; Ricci et al., 2020). A total of 897 participants (WB-EMS: *n* = 565, control group: *n* = 332) took part in the included studies. Sample size in the study arms ranged from 10 (Bellia et al., 2020; Ricci et al., 2020) to 134 participants (Vatter, 2010) in the WB-EMS group and from 10 (Vatter, 2010; Ricci et al., 2020) to 38 (TEST III-study; Kemmler et al., 2014, 2015) participants in the control group (CG). The mean age ranged from 23 (Jee, 2019) to 77 years (FranSO-study; Kemmler et al., 2018a,b), FORMOsA-study (Kemmler et al., 2016c,d), and the mean body mass index (BMI, kg/m^2^) varied from 22 (Jee, 2019) to 38.2 kg/m^2^ (Ricci et al., 2020). Twelve RCTs were conducted in Germany (Kemmler et al., 2010a,b, 2014, 2016c,d, 2018a,b; Vatter, 2010; Kemmler and von Stengel, 2013; Schink et al., 2018; Weissenfels et al., 2018; Ludwig et al., 2019), two in Korea (Jee, 2019; Kim and Jee, 2020), one in Italy (Bellia et al., 2020), and one in Brazil (Ricci et al., 2020). One trial implemented two EMS groups with varying impulse frequency (Ludwig et al., 2019). Another study evaluated three WB-EMS groups with different impulse intensities (Jee, 2019). Six trials recruited female participants (Kemmler et al., 2010b, 2014, 2016c,d; Kemmler and von Stengel, 2013; Kim and Jee, 2020), four studies focused on male subjects (Kemmler et al., 2010a, 2018a,b; Jee, 2019), and six studies included both genders in their interventions (Vatter, 2010; Schink et al., 2018; Weissenfels et al., 2018; Ludwig et al., 2019; Bellia et al., 2020; Ricci et al., 2020). Three studies included people with low muscle mass/sarcopenia (i.e., TEST III, FORMOsA, FranSO), five studies focused on people with obesity and/or cardiometabolic diseases (Kemmler et al., 2010a; Kemmler and von Stengel, 2013; Bellia et al., 2020; Kim and Jee, 2020; Ricci et al., 2020), one study each included low back patients (Weissenfels et al., 2018) or advanced cancer patients (Schink et al., 2018). All the other studies focused on healthy, untrained to moderately trained adults.

### Study Interventions

While most of the studies applied isolated WB-EMS as the primary study intervention, two studies combined WB-EMS with either advanced nutritional support in cancer patients (Schink et al., 2018) or an energy-restricting diet (600 kcal/d) in people with the metabolic syndrome. Five studies provided adjuvant whey protein [(FORMOsA; Kemmler et al., 2016c,d), FranSO (Kemmler et al., 2018a,b); Schink et al. (2018) and/or Test I (Kemmler et al., 2010b, 2015), TEST III (Kemmler et al., 2014, 2015), TEST IIIsub (Kemmler and von Stengel, 2013)], cholecalciferol supplementation (800–1,000 IE/d) for both groups according to recent recommendations (DGE (German Nutrition Society), 2012; Bauer et al., 2013) (Table 1).

**Table 1:** Study and intervention characteristics of the included studies (*n* = 16).

**References**	**Study design**	**Sample size (*n*)**	**Status**	**Sex, Age MV ± SD**	**Control group**	**Intervention**	**Main outcomes**	**Comment**
Bellia et al. (2020)	RCT, parallel group	EMS: 10 CG: 11	MetS, Obesity, physically active, untrained	♀ + ♂ 50 ± 7 yrs.	Diet (caloric restriction −600 kcal/d)	Combined diet (-600 kcal/d) and WB-EMS. 1-3x 20 min/week (first 12 weeks), 2 × 20 min/w (last 14 weeks) for 26 weeks (Actiwave, Gyor, Hungary): 15 min, **85 Hz, 400** **μs, 0.3 s ramp**, 4s−4s, 5 min **14 Hz**, 10 s – 15 s, intensity: RPE 6-7 (Borg CR10), active EMS with movements with small tools (e.g., elastic bands).	Body composition, cardiometabolic risk factors	Energy restriction in both groups
Jee (2019)	RCT, parallel group	L-Int: 13 M-Int: 14 H-Int: 14 CG: 13	Healthy, physically active, untrained	♂ 20–29 yrs.	Same exercises without EMS (placebo)	3× 20 min/week for 6 weeks (Miracle, Seoul, Korea), **85 Hz, rectangular, 350** **μs**, 6-4s, active WB-EMS with 10 moderate intense isometric exercises, intensity: 50% (L-Int) vs. 60% (M-Int) vs. 80% of maximum impulse tolerance (MT) (H-Int).	Body composition, adipokines, leg strength (KE)	Dose response study for “intensity”
Kemmler et al. (2010b, 2015)	RCT, parallel group	15/15	Healthy, moderately trained	♀ 66 ± 6 yrs.	Maintain exercise	1.5 sessions/w., 14 w (miha bodytec, Gersthofen, Germany), 10 min **bipolar, rectangular, 350** **μs**; 10 min with **85 Hz**. 4s impulse - 4s rest, and 10 min with **7 Hz. continuous impulse**; intensity: RPE 6–7 (hard+ to very hard; Borg CR-10), active WB-EMS with 1–2 sets, 10 exercises, 6-8 reps during impulse phases in a standing position.	Body composition, leg strength (LP), trunk strength, RMR	
Kemmler et al. (2010a, 2015)	RCT, parallel group	14/14	MetS, physically active, untrained	♂ 69 ± 3 yrs.	Semi-active: Low-intensity whole-body vibration	1.5× 25 min/w.; 14 w (miha bodytec), 10 min on cross-trainer (70% VO_2_peak), **bipolar, rectangular, 85 Hz, 350** **μs**; continuous impulse and 15 min active WB-EMS with 2 sets, 7 exercise, 6–8 reps, **bipolar, rectangular, 85 Hz, 350** **μs**, 4s – 4 s; intensity: RPE 6-7 (hard+ to very hard, Borg CR10).	Body composition, leg strength (LP)	
Kemmler et al. (2014, 2015)	RCT, parallel group	38/38	Osteopenia, low muscle mass untrained	♀ 75 ± 5 yrs.	Semi-active: wellness	1.5× 20 min/w., 12 months (miha bodytec), **bipolar, rectangular, 85 Hz, 350** **μs**, 4−6 s impulse – 4 s rest; intensity: RPE 6–7 (hard+ to very hard, Borg CR-10); 1–2 sets of 8-12 movements with 6–8 reps during the impulse phases; CG: 2× 10 weeks with one session/w. low-intensity exercises for well-being	Body composition, BMD, leg strength (LP)	
Kemmler and von Stengel (2013)	RCT, parallel group	23/23	See above, +abdomin. obesity	♀ 75 ± 5 yrs.	Semi-active: wellness	See (Kemmler et al., 2014, 2015), however, only subjects with waist circumferences >80 cm were included	Total and regional body composition, leg strength (LP)	Sub-analysis TEST III-study
Kemmler et al. (2016c,d)	RCT, parallel group	25/25	Sarcopenic Obesity, physically active, untrained	♀, 77 ± 4 yrs.	Inactive	1× 20 min/w., 26 weeks (miha bodytec)**, bipolar, rectangular, 85 Hz, 350** **μs**, 4-6 s impulse – 4 s rest; intensity: RPE 5-6 (hard-hard+, Borg CR-10), active WB-EMS with 1-2 sets of 12 low intense movements during the impulse phase. with 6-8 reps in a supine position	Body compos. leg (LP) and back strength, MetS	
Kemmler et al. (2017b, 2018a)	RCT, parallel group	33/34	Sarcopenic Obesity, physically active, untrained	♂, 77 ± 5 yrs.	Inactive	1.5× 20 min/w., 16 weeks (miha bodytec)**, bipolar, rectangular, 85 Hz, 350** **μs**, 6 s impulse – 4 s rest; intensity: RPE 6–7 (hard+ to very hard, Borg CR-10), active WB-EMS: 1–2 sets of 12 movements with 6–8 reps during impulse phases.	Total/ regional body composition, leg (LP) and trunk strength, MetS, Renal function	
Kim and Jee (2020)	RCT, parallel group	15/15	Obesity, physically active, untrained	♀, 71 ± 3 yrs.	Same exercises without EMS (placebo)	3× 40 (?) min/w. for 8 weeks (Miracle, Seoul, Korea), **bipolar, rectangular**, **85 Hz, 350** **μs**, 6–4s, intensity: 60–80% MT, active WB-EMS during (aerobic) exercise with music	Body composition, biomarkers	Adjuvant moderate intensity exercise
Ludwig et al. (2019)	RCT, parallel group	20 Hz: 19 85 Hz: 19 CG: 15	Healthy, physically active, Untrained	♀ + ♂ 25 ± 4 yrs.	Inactive	1.5× 20 min/w., for 10 weeks (miha bodytec), **bipolar, 20 “vs.” 85 Hz, 350** **μs**, 4–4s, rectangular, intensity: RPE: 6–7, active WB-EMS with 9 low intensity movements/exercises, 12–15 reps (partially unilateral) during impulse phases.	Trunk-strength, -power, body posture	Dose-response study: 20 vs. 85 Hz.
Ricci et al. (2020)	RCT, parallel group	10/10	Obesity, Bariatric surgery untrained	♀ + ♂ 18–50 yrs.	Same exercises without EMS (placebo)	5× 12-15 min/w., 6 weeks (miha bodytec), **bipolar, 85 Hz, 350** **μs**, 6 s impulse – 4 s rest, 3× 15 min/w. and **30 Hz, 350** **μs**, 4–10 s, 2× 12 min/w.; intensity n.g., active WB-EMS with 10 low intensity exercises during impulse phases.	Body composition, endurance	WB-EMS after bariatric surgery
Schink et al. (2018)	NCT, parallel group	96/35	Advanced tumor Sedentary untrained	♀ + ♂ 60 ± 13 yrs.	Inactive	2× 20 min/w., 12 weeks (miha bodytec), **bipolar, 80 Hz, 350** **μs**, **rectangular**, 6 s impulse – 4 s rest; intensity: RPE 5-6, active WB-EMS with 7 low-intensity movements, 2 sets, 8-12 reps during impulse phases.	Body composition, endurance, renal function	
Vatter (2003, 2010)	NCT, parallel group	134/10	Healthy, trained	♀ + ♂ 43 ± 12 yrs.	Maintain exercise	2× 45 min/w., 6 weeks (body transformer, GPL-Concept, Germany), **bipolar, 85 Hz**, **rectangular, 350** **μs**, 4s impulse - 4s rest, intensity: RPE at threshold tolerance; 12 isometric exercises.	Body composition, upper back strength, low back pain	
Weissenfels et al. (2018)	RCT, parallel group	15/15	Back pain, physically active, untrained	♀ + ♂ 40–70 yrs.	Inactive	1× 20 min/w., 12 weeks (miha bodytec), **bipolar, 85 Hz, 350** **μs, rectangular**, 6s−4 s, intensity, RPE: 5–7 (hard-very hard, Borg CR10), 6 low-intensity back specific movements, 2–3 sets, 6 reps during impulse phases	Trunk strength, low back pain	

*CG, Control group; KE, Knee extension; LP, Leg press; L-, M-, H-Int, low-, moderate-, high-intensity; MetS, Metabolic Syndrome; MT, Maximum impulse tolerance; NCT, Non-randomized controlled trial; n.g., not given; RPE, Rate of received exertion; RCT, Randomized controlled trial, RT, Resistance training. Bold: Impulse parameters of the WB-EMS protocol*.

### WB-EMS Protocol

The WB-EMS protocols (i.e., impulse parameters) were quite homogeneous between the studies. All studies applied bipolar, low frequency protocols of 80–85 Hz [apart from one study arm that prescribed 20 Hz (Ludwig et al., 2019)] with a rectangular pulse wave form (Bellia et al., 2020: 0.3 s ramp). Impulse width was specified at 350 or 400 μs (Bellia et al., 2020). Apart from one study that applied a longer impulse break (10 s; Ricci et al., 2020), all the studies used intermitted protocols with 4–6 s of impulse and 4 s of impulse break. However, two studies structured their WB-EMS sessions in two parts with either an intermitted and a continuous WB-EMS sequence (10 min with 7 Hz.) continuous impulse; TEST I: Kemmler et al., 2010b, 2015) or an intermitted protocol with shorter (15 min 4 s−4 s) and longer impulse phases (5 min 10–15 s). With two longer (Vatter, 2010: 45 min; Kim and Jee, 2020: 40 min^5^) and one shorter exceptions (12–15 min, Ricci et al., 2020), all the other WB-EMS protocols averaged 20–25 min/session. All studies combined WB-EMS with voluntary movements or isometric exercises (Vatter, 2010; Jee, 2019) with negligible (FORMOSA; Kemmler et al., 2016c,d), low (TEST I, TEST III; TEST IIIsub, FranSO, Kemmler et al., 2010a,b, 2014, 2015, 2017b, 2018a; Vatter, 2010; Schink et al., 2018; Ludwig et al., 2019; Ricci et al., 2020), or moderate intensity (Jee, 2019; Bellia et al., 2020; Kim and Jee, 2020) during the impulse phase. Impulse intensity of the WB-EMS application as predominately prescribed by rating of perceived exertion (Borg CR 10; Borg and Borg, 2010) consistently averaged hard (Borg 5) to very hard (Borg 7). One working group (Jee, 2019; Kim and Jee, 2020), however, used an approach that was based on maximum impulse tolerance (MT)^6^ and applied three different intensities (i.e., 50, 60, and 80% 1MT) (Table 1).

The weekly training frequency was less homogeneous, ranging from one session (Weissenfels et al., 2018) to five sessions per week (Ricci et al., 2020) and weekly training volume ranging from 20 (Weissenfels et al., 2018) to 120 min/week (Kim and Jee, 2020). Length of the WB-EMS trials varied from 6 weeks (Vatter, 2010; Jee, 2019; Ricci et al., 2020) to 12 months (Kemmler et al., 2014, 2015) (Table 1).

Of importance, although not consistently stated, none of the studies reported negative side effects of WB-EMS applications.

### Control Group

Six studies implemented a physically inactive control group (Kemmler et al., 2016c,d, 2017b, 2018a; Schink et al., 2018; Weissenfels et al., 2018; Ludwig et al., 2019; Bellia et al., 2020), while in two studies exercise habits (resistance exercise) were maintained (Vatter, 2003; Kemmler et al., 2010b, 2015). Three studies applied the same movements/exercises provided during the WB-EMS condition albeit with the electric current turned off (Table 1). The remaining three trials (Kemmler et al., 2010a, 2014, 2015; Kemmler and von Stengel, 2013) implemented semi-active control groups that performed supervised exercises with no or minor impact (i.e., “sham exercise”) on the outcomes addressed here (Table 1).

### Assessments of Study Outcomes

**Figure 2:**
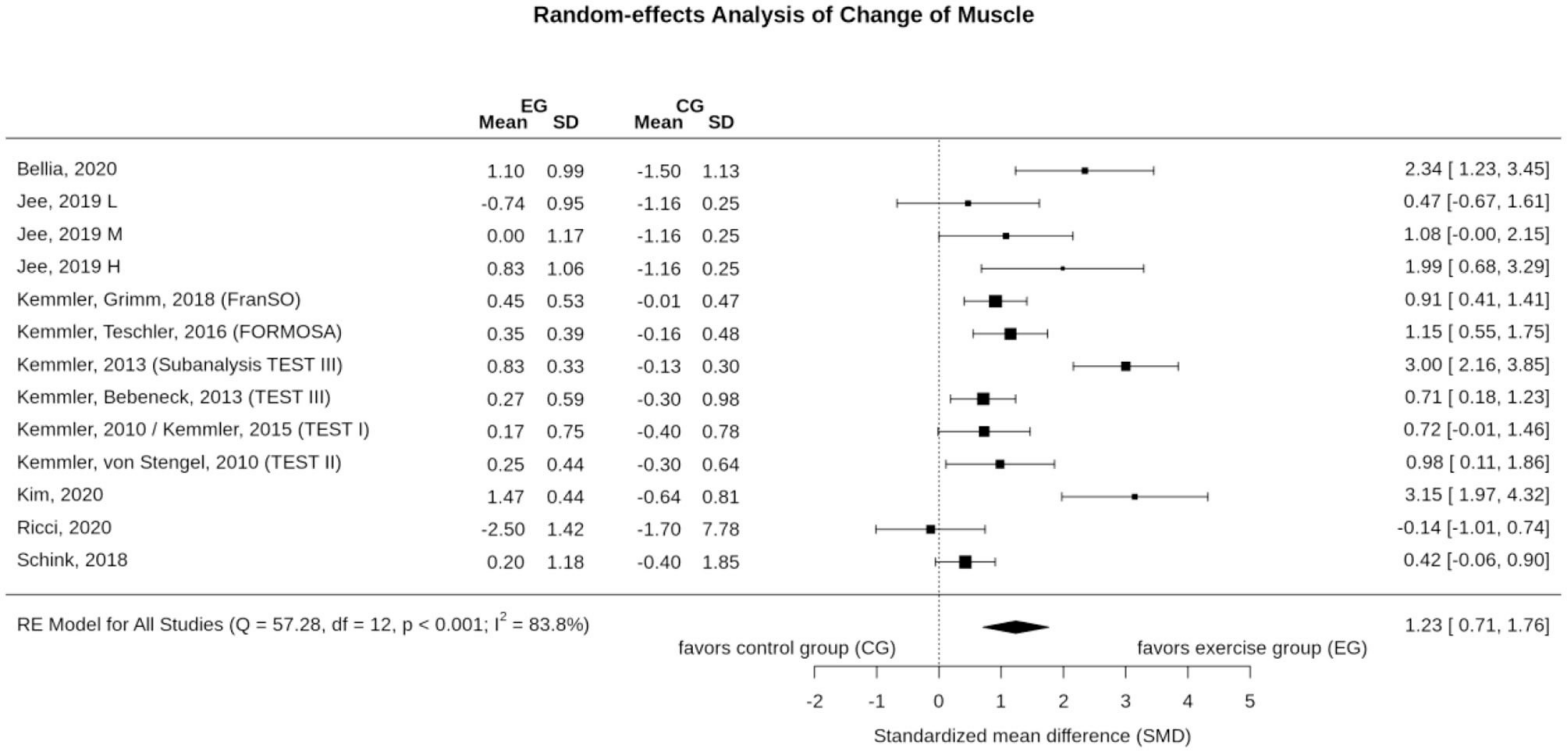
Forest plot of meta-analysis results on muscle mass. The data are shown as pooled standard mean differences (SMD) with 95% CI for changes in WB-EMS and control groups.

In summary, 14 study arms focused on WB-EMS effects on body composition (Figures 2, 4). Nine study arms determined body fat and/or fat free mass via direct-segmental multi-frequency bio-impedance analysis (DSM-BIA) (Vatter, 2003; Kemmler et al., 2018a; Schink et al., 2018; Jee, 2019; Bellia et al., 2020; Kim and Jee, 2020; Ricci et al., 2020); four studies (TEST II, TEST III; TEST IIIsub, FORMOsA; Kemmler et al., 2010a, 2014, 2016c,d; Kemmler and von Stengel, 2013) applied dual-energy x-ray absorptiometry, and one study (Kemmler et al., 2010b, 2015) used the caliper method (Durnin and Womersley, 1974) and indirect calorimetry to determine fat free body mass. Five study groups reported data on lean body mass/fat free mass (Kemmler et al., 2014, 2015; Bellia et al., 2020; Ricci et al., 2020), three studies with five WB-EMS groups published data on skeletal muscle mass (Schink et al., 2018; Jee, 2019; Kim and Jee, 2020), and four studies (Kemmler et al., 2010a, 2014, 2016b, 2018a) reported data on appendicular skeletal muscle mass (ASMM; i.e., lean body mass of the upper and lower limbs).

In summary, six studies determined leg extension strength. Five studies applied either isometric (TEST I, TEST III, TEST IIIsub) or isokinetic leg-press exercise (FORMOsA, FranSO), a further study (Jee, 2019) focused on isokinetic knee extension exercise to evaluate maximum knee extension strength. Lastly, five studies/study arms (Kemmler et al., 2010b, 2014, 2015; Weissenfels et al., 2018; Ludwig et al., 2019) determined maximum (isometric) trunk extension strength. Two studies (TEST I, TEST III) assessed trunk extension strength in a sitting position with slightly bent (10°) upper body, three other studies/study arms (Weissenfels et al., 2018; Ludwig et al., 2019) evaluated this parameter in a standing upright position (0°).

### Risk of Bias Assessment of the Included Studies

Risk of bias assessments are shown in Table 1. According to the PEDro scale (Sherrington et al., 2000; de Morton, 2009), the majority of included studies had a high methodological quality (Kemmler et al., 2010a, 2014, 2015, 2016c,d, 2018a,b; Weissenfels et al., 2018; Ludwig et al., 2019; Kim and Jee, 2020; Ricci et al., 2020). Two studies were graded as moderate methodological quality studies (Kemmler et al., 2010b; Jee, 2019).

The overall methodological quality of the included studies as assessed by the TESTEX scale (Smart et al., 2015) was rated from six to 15 out of 15 points. Of importance, since TESTEX is largely based on PEDro, we focus on the exercise specific issues and overall TESTEX score in Table 1. Two studies achieved full points (Kemmler et al., 2018b; Weissenfels et al., 2018). Eight trials achieved a score of between 12 and 14 (Table 1) and three trials were rated as having 10 and 11 points (Jee, 2019; Ludwig et al., 2019; Kim and Jee, 2020). The remaining studies were scored with seven to nine points (Vatter, 2010; Schink et al., 2018; Bellia et al., 2020).

### Effect of WB-EMS on Muscle Mass

Eleven studies with 13 WB-EMS groups evaluated the effect of WB-EMS on muscle mass (Figure 2). In summary, the exercise intervention resulted in significant effects (1.23; 95%-CI: 0.71–1.76), albeit with a substantial level of heterogeneity between the trials (*I*^2^ = 83.8%, *Q* = 57.3) (Figure 2). Sensitivity analysis revealed the most similar effect when the mean correlation coefficient was utilized to impute SD of the absolute change for those studies with missing SDs, and when the analysis was computed among studies with available SDs of the change. However, imputing minimum (SMD: 1.88, 95%-CI: 1.04–2.72) or maximum SD (SMD: 1.11, 95%-CI: 0.61–1.60) resulted in similar significant results.

**Figure 3:**
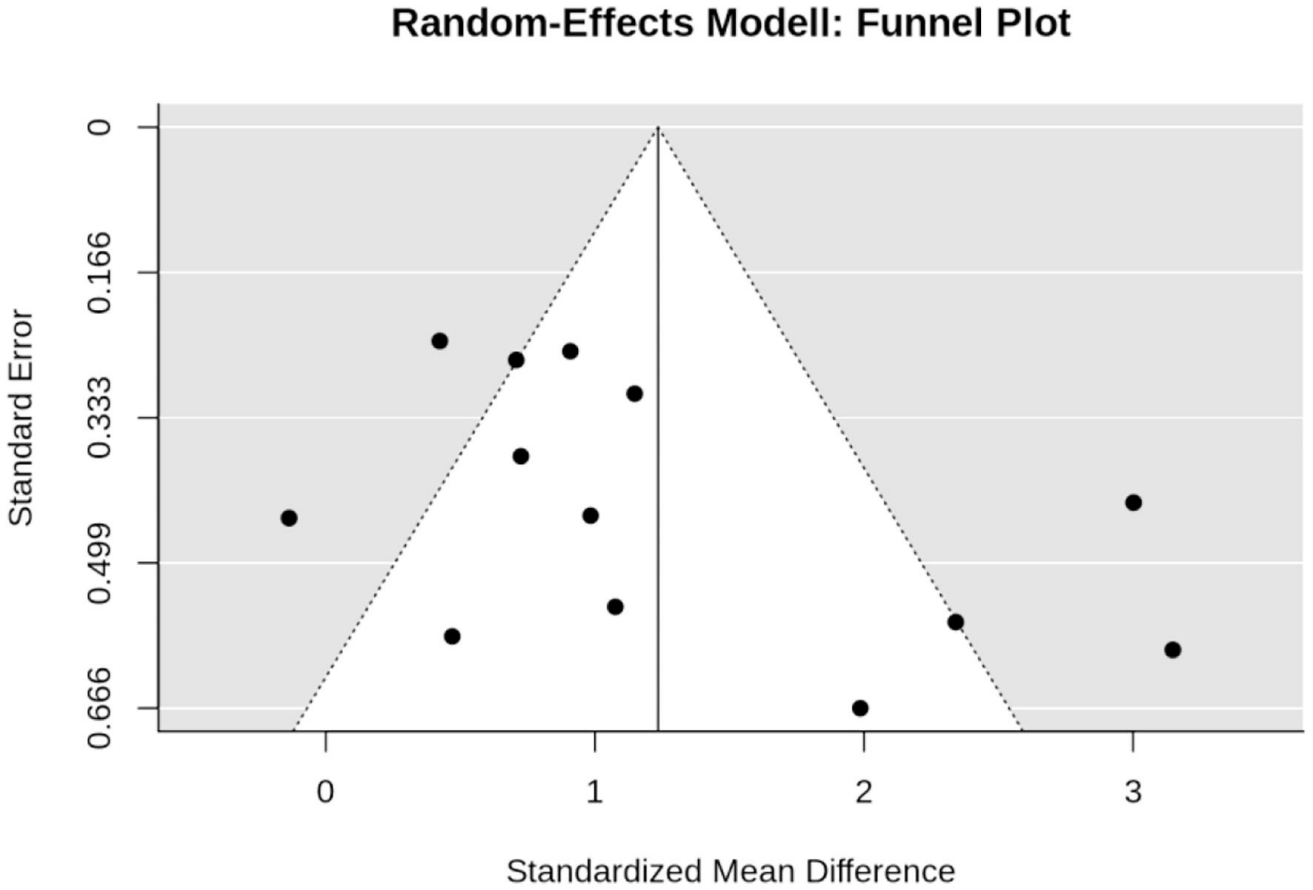
Funnel plot of the WB-EMS studies that addresses muscle mass.

In summary, the funnel plot did not provide evidence for a small-study bias (Sterne et al., 2011) (Figure 3). The regression (*p* = 0.085) and the rank (*p* = 0.359) correlation test for funnel plot asymmetry did not indicate significant asymmetry (Figure 3).

### Effect of WB-EMS on Total Body Fat Mass

**Figure 4:**
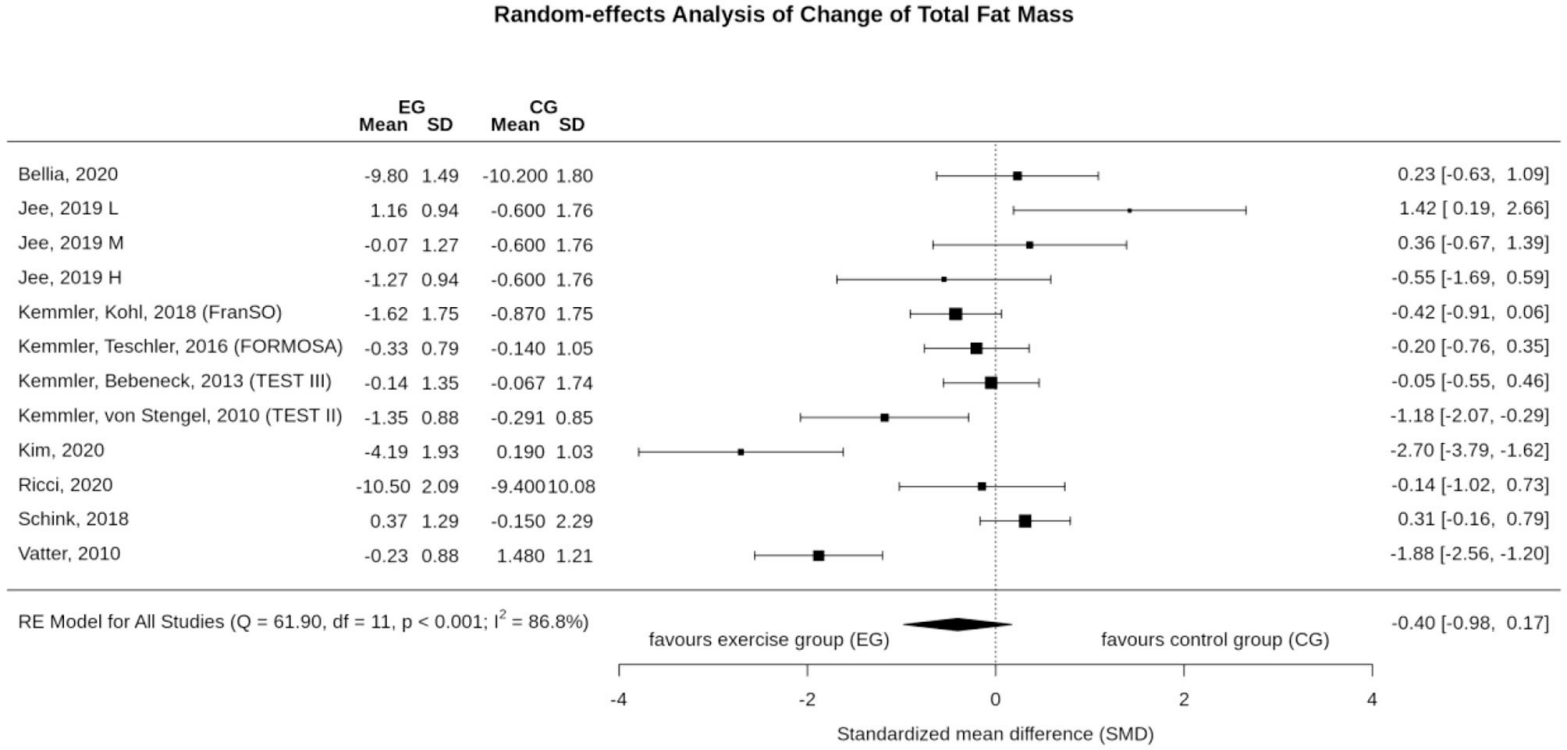
Forest plot of meta-analysis results on total body fat. The data are shown as pooled standard mean differences (SMD) with 95% CI for changes in WB-EMS and control groups.

Ten studies with 12 study groups determined the effect of WB-EMS on total body fat mass (Figure 4). In summary, WB-EMS did not significantly affect (*p* = 0.170) total body fat mass. The pooled estimate of random effect analysis was SMD −0.40, 95%-CI: 0.17–0.98. We observed a substantial level of heterogeneity between the trials (*I*^2^ = 86.8%, *Q* = 61.9) (Figure 4). Sensitivity analysis revealed the most similar effect when the mean correlation coefficient was utilized to impute SD of the absolute change for those studies with missing SDs, and when the analysis was computed among studies with available SDs of the change. However, imputing minimum or maximum SD resulted in similar non-significant results.

**Figure 5:**
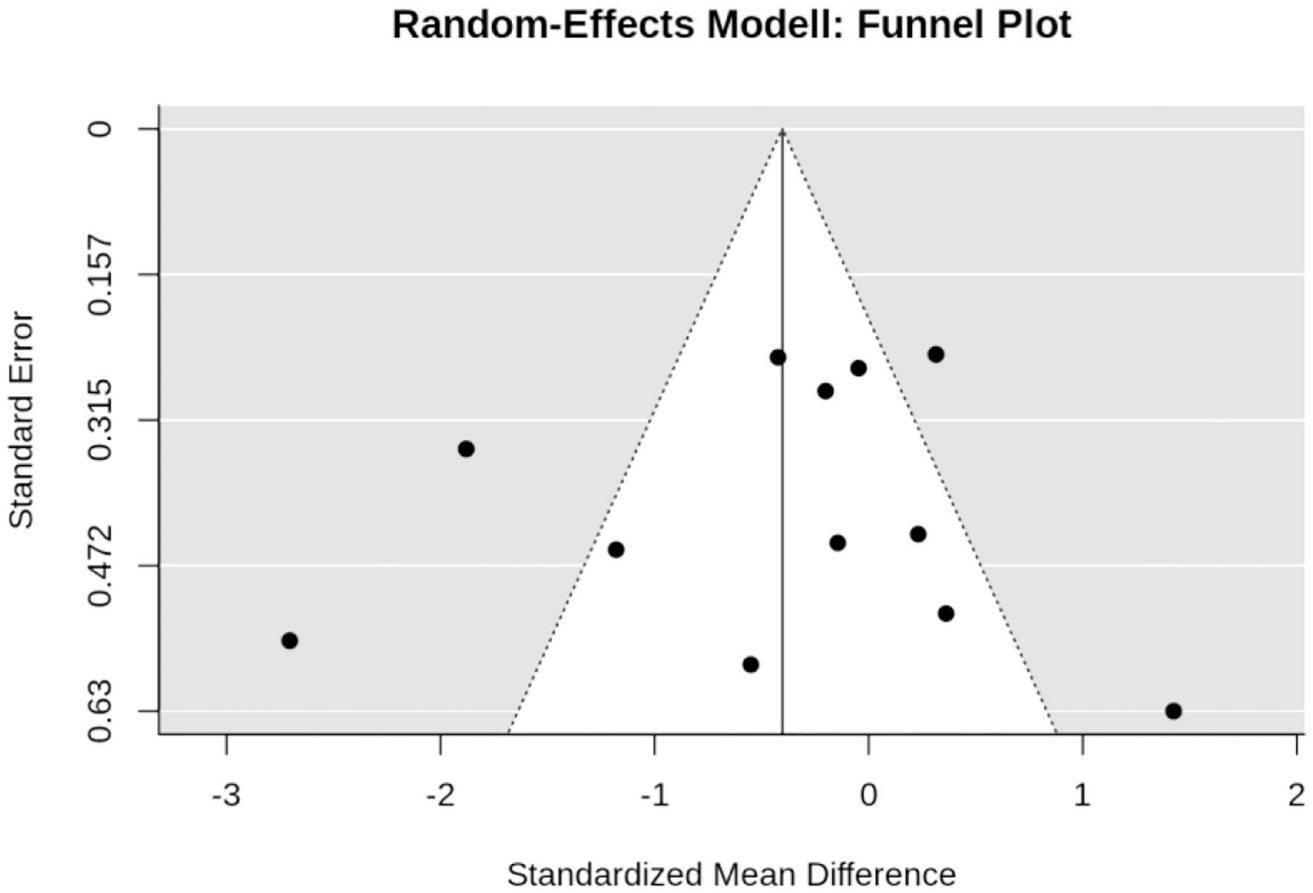
Funnel plot of the WB-EMS studies that addresses total body fat mass.

Figure 5 shows the funnel plot on WB-EMS and total body fat mass effects that did not provide evidence for a significant small-study bias. Regression (*p* = 0.58) and rank test (*p* = 0.84) for funnel plot asymmetry were non-significant.

### Effect of WB-EMS on Maximum Leg Extensor Strength

**Figure 6:**
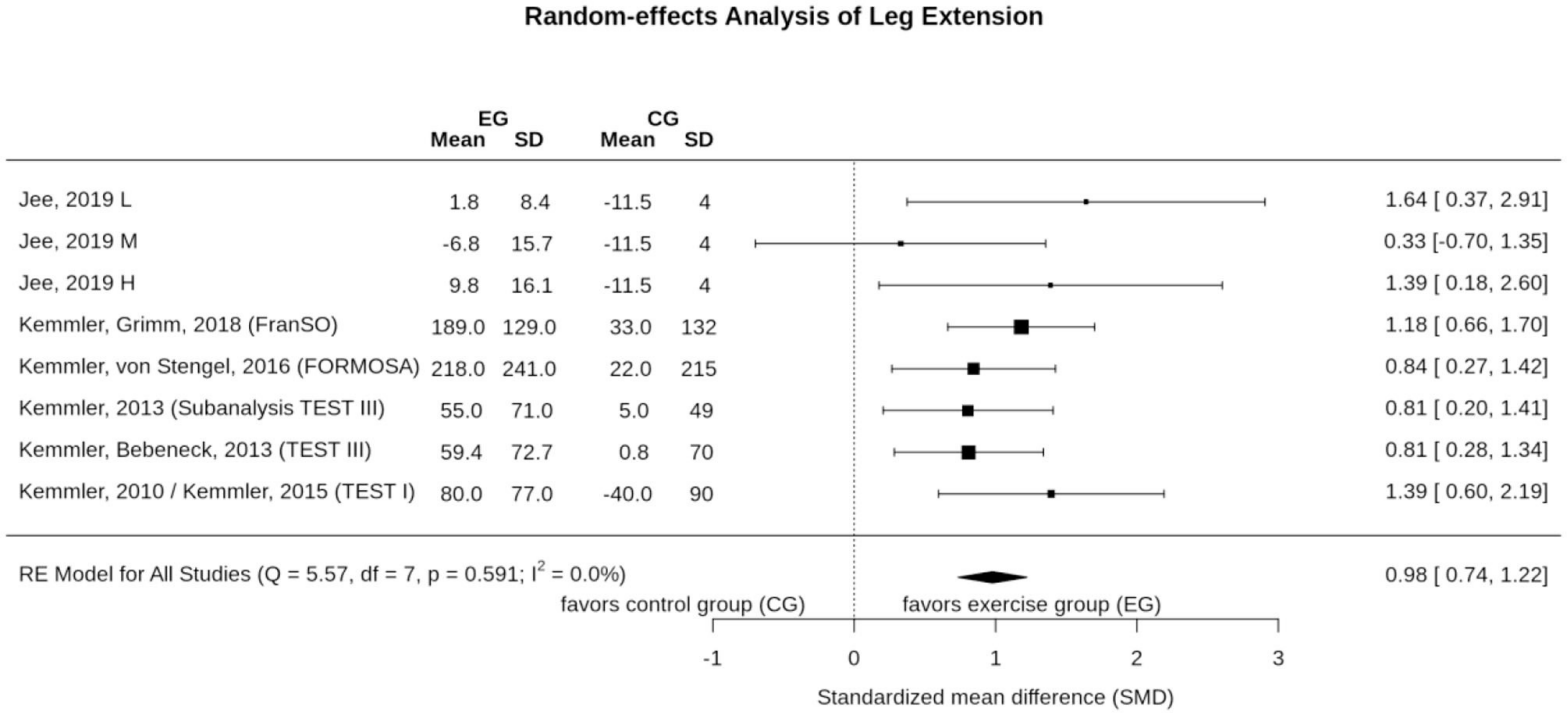
Forest plot of meta-analysis results on maximum leg extension strength. The data are shown as pooled standard mean differences (SMD) with 95% CI for changes in WB-EMS and control groups.

Six studies with eight study arms evaluated the effect of WB-EMS on maximum leg extensor strength (Figure 6). In summary, we observed a significant effect (*p* < 0.001) of WB-EMS on maximum leg extensor strength. SMD for the effect size was 0.98 with a 95%-CI of 0.74–1.22. Q (5.6) and *I*^2^- statistics (0.0%) revealed no significant (*p* = 0.591) heterogeneity between the trials. Sensitivity analysis revealed the most similar effect when the mean correlation coefficient was utilized to impute SD of the absolute change for those studies with missing SDs, and when the analysis was computed among studies with available SDs of the change. Sensitivity analysis with imputation of minimum (1.19, 95%-CI: 0.81–1.57) or maximum (0.92, 95%-CI: 0.68–1.16) SDs resulted in similar significant effects.

**Figure 7:**
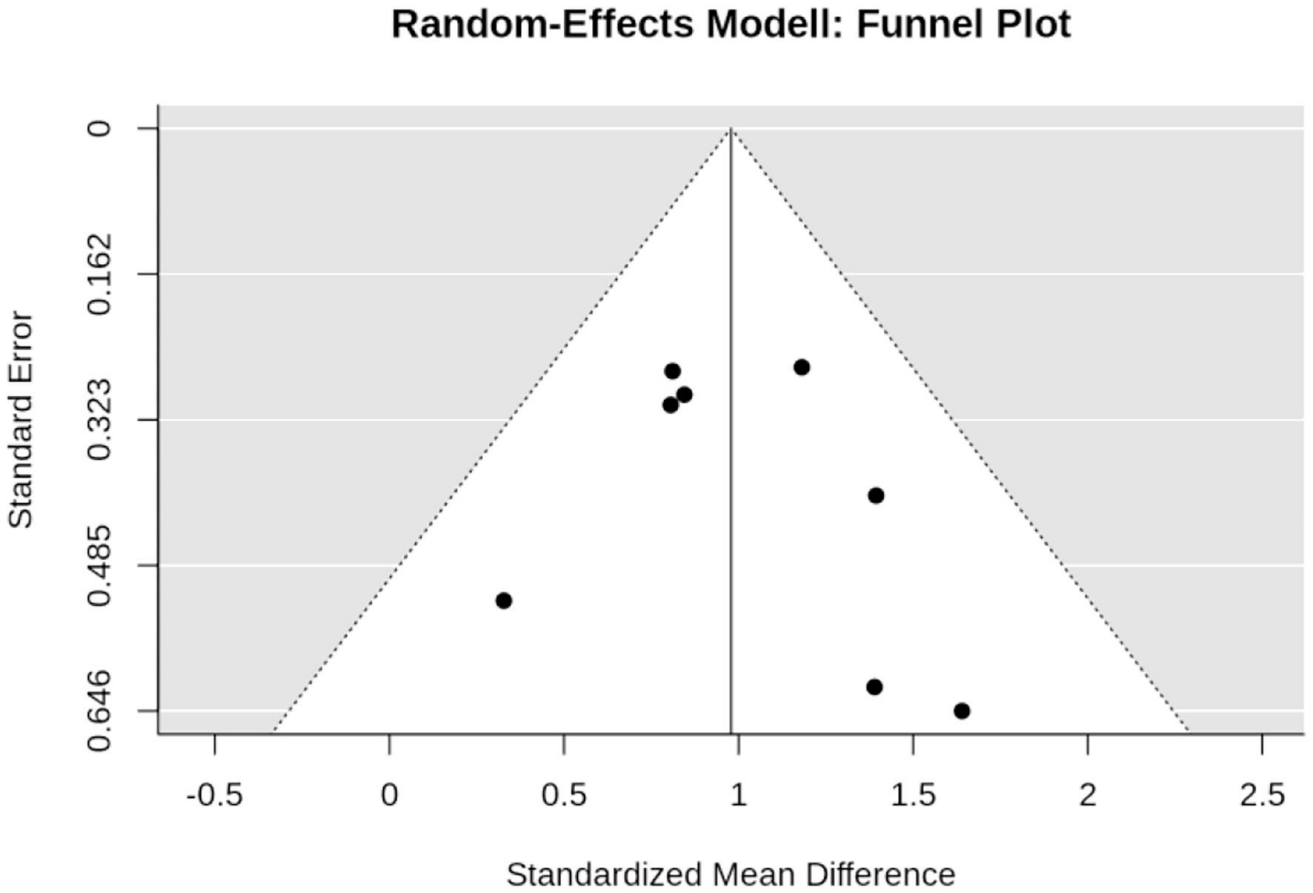
Funnel plot of the WB-EMS studies that addresses maximum leg extension strength.

Figure 7 shows the funnel plot for WB-EMS studies that referred to maximum leg extension strength. In summary, the funnel plot did not provide evidence for a small-study bias; furthermore; neither the regression test (*p* = 0.49) nor the rank test (*p* = 0.72) indicates asymmetry.

### Effects of WB-EMS on Maximum Trunk Extension Strength

**Figure 8:**
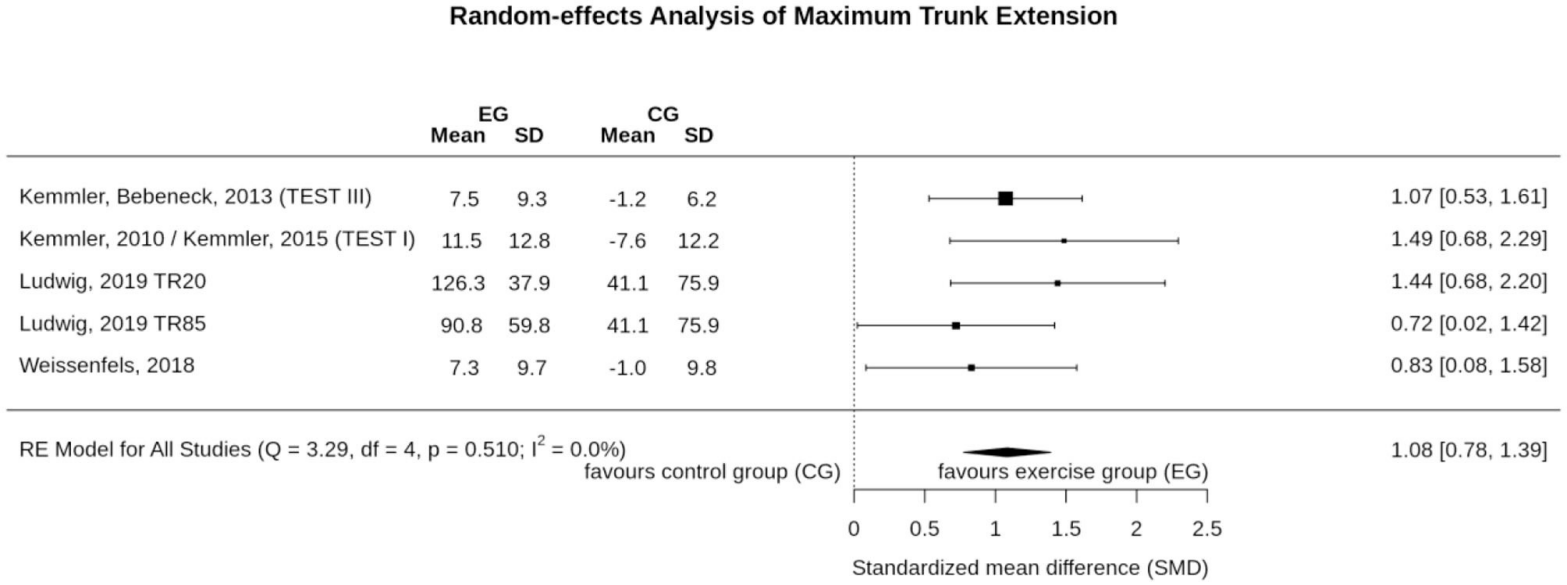
Forest plot of meta-analysis results on maximum trunk extension strength. The data are shown as pooled standard mean differences (SMD) with 95% CI for changes in WB-EMS and control groups.

Four studies with five study arms determined the effect of WB-EMS on maximum trunk extension strength (Figure 8). In summary, the WB-EMS intervention resulted in significant positive effects (*p* < 0.001) with pooled estimate of random effect analysis (SMD) of 1.08, 95%-CI: 0.78–1.39. Q (3.3) and *I*^2^- statistics (0.0%) indicate no significant (*p* = 0.510) heterogeneity between the trials. Sensitivity analysis revealed the most similar effect when the mean correlation coefficient was utilized to impute SD of the absolute change for those studies with missing SDs, and when the analysis was computed among studies with available SDs of the change. Imputing minimum or maximum SD resulted in similar results.

**Figure 9:**
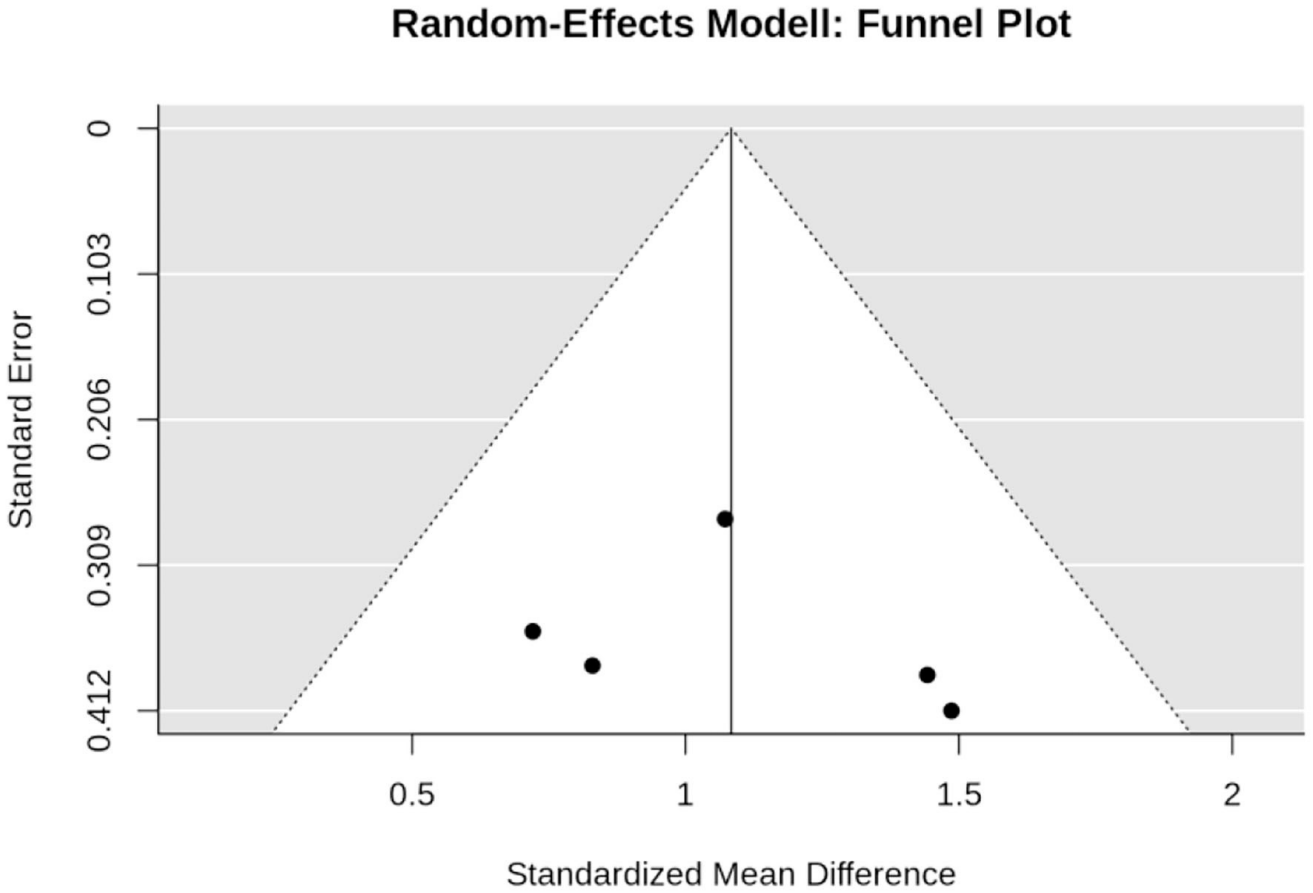
Funnel plot of the WB-EMS studies that addresses maximum trunk extension strength.

Funnel plot (Figure 9), regression test (*p* = 0.631), and rank test (*p* = 0.233) did not indicate positive evidence for a small-study bias or significant asymmetry in general.

## Discussion

In this meta-analysis, we clearly determined the significant favorable effects of WB-EMS on muscle mass and strength, but not on total body fat. Considering our eligibility criteria and the corresponding cohorts included, we thus provided final evidence for the significance of WB-EMS on body composition and muscle strength in predominately untrained, middle-aged to older adults.

### Effect of WB-EMS Application on Total Body Fat Mass

The result on the missing significant WB-EMS-induced body fat mass is contrary to our hypothesis since fat reduction exceeded lean body mass gains after WB-EMS application in some cases (Kemmler et al., 2016b, 2017a). Reviewing our data (Table 1, Figure 4), some study characteristics and features might have contributed to this result. Of note, three out of 12 WB-EMS study groups that reported total body fat mass data determined the effect of WB-EMS after bariatric surgery (Ricci et al., 2020), during moderate (600 kcal/d) caloric restriction (Bellia et al., 2020) or during nutritional support (Schink et al., 2018) of advanced cancer therapy.^7^ In the two studies that aimed to reduce body fat mass, reduction averaged about 10 kg after 6 (Ricci et al., 2020) or 26 weeks (Bellia et al., 2020) with minor differences between WB-EMS and CG. Thus, due to the outstanding effect of caloric restriction/bariatric surgery, the less prominent contribution of WB-EMS on body fat mass reductions might have been masked. In parallel, in their cohort of cancer patients, Schink et al. (2018) focused on increases of caloric intake that might also have confounded the effect of WB-EMS on body fat reduction. Of interest, there is some evidence for a dose/response effect of impulse intensity that impacts body fat more prominently than muscle mass changes (Jee, 2019). However, the significant negative effect of low impulse intensity on total fat mass in the absence of confounding factors reported in this study (Jee, 2019) remains to be replicated. On the other hand, the outstanding group differences (4.4 kg; SMD: 2.70 Figure 3) in favor of the WB-EMS group (Figure 4) after eight weeks of exercise with music and with or without WB-EMS (40 min/session)^8^ in the absence of any changes of caloric intake (…or output) in the WB-EMS or CG, reported by the same research group (Kim and Jee, 2020) is surprising. Revisiting energy expenditure as one main determinant of weight reduction, much like resistance exercise, WB-EMS acts via three pathways: (1) the acute WB-EMS effect (Kemmler et al., 2012; Boccia et al., 2017), (2) post-exercise regeneration and adaptation (Teschler et al., 2018), and (3) increases in resting metabolic rate (RMR) due to increases in muscle mass (Aristizabal et al., 2015). However, all options are related to adequate (high) impulse intensity and—for the latter determinant—longer training periods. However, the majority of the WB-groups studies (Vatter, 2010; Jee, 2019; Kim and Jee, 2020; Ricci et al., 2020) applied WB-EMS applications for 6–8 weeks, thus the RMR effect on energy expenditure was less pronounced. Summing up, there are several study characteristics and aspects that might have confounded the proper effect of WB-EMS on total body fat reduction, and hence our result on body fat changes should be treated with care.

### Effect of WB-EMS Application on Muscle Mass

Although the favorite training aim of many WB-EMS applicants/clients is fat reduction, its effect on muscle mass and strength is the more evident research issue given the nature of WB-EMS as a resistance type intervention. In summary, we observed significant results, with large effect sizes in favor of WB-EMS particularly on muscle mass parameters (SMD: 1.23). One may argue that the same confounders addressed for total fat mass might impact the results on muscle mass and strength. Indeed, as for total body fat mass, Q and *I*^2^ statistics revealed substantial heterogeneity between the trial results for muscle mass (but not for muscle strength). Reviewing the individual study results, the same studies prominent in fat mass changes are striking for muscle mass changes. For example, Bellia et al. (2020), who applied WB-EMS during energy restriction (−600 kcal/d) for 26 weeks (Table 1), reported very positive results on muscle mass. In a recent study not included here,^9^ Willert et al. (2019) focused (also) on the maintenance of muscle mass during 16 weeks of negative energy balance (-500 kcal/d) in overweight-obese premenopausal women applying WB-EMS and protein supplementation. Leaving aside differences with respect to study duration (16 vs. 26 weeks) and weekly WB-EMS volume (30 vs. 40 min), the WB-EMS protocol and assessment (DSM-BIA, InBody 770, Seoul Korea) of both studies were largely comparable, but the effect sizes determined by Willert et al. (2019) were much lower compared to the study of Bellia et al. (2020) (SMD: 2.34 vs. 0.27). In contrast, the study of Ricci et al. (2020), which also focused on weight reduction, albeit by bariatric surgery, was the only study to report tendentially negative effects of WB-EMS on muscle mass. Further, one may rightly argue that the effect of (whey) protein supplementation (however for WB-EMS and CG) as applied in three further studies (FORMOsA, FranSO; Kemmler et al., 2016c,d, 2017b, 2018a; Schink et al., 2018) might produce a synergistic effect (Bauer et al., 2013; Lancha et al., 2017) and thus impact the proper group comparison. Lastly, one may criticize that studies that determined ASMM might achieve suboptimal results, an outcome that does in fact indicate an even higher effect than determined by these studies, due to the higher amount of electrode area placed at the trunk compared with the extremities. Apart from the potentially confounding parameters, there is also evidence for a dose response effect of impulse intensity (Jee, 2019), with higher muscle mass gains with higher impulse intensity, a link that cannot be confirmed for maximum leg extensor strength (Figure 6), however.

### Effect of WB-EMS Application on Maximum Strength

Another study result that should be addressed in more depth is the lower muscle strength compared to muscle mass changes observed in this meta-analysis. A corresponding issue frequently covered is the low functionality of WB-EMS *per se* (Seyri and Maffiuletti, 2019). However, all the included studies applied a combined protocol that used WB-EMS as the main physical intervention and added voluntary muscle activation by dynamic movements/exercises. In a recent study with older women (Kemmler et al., 2015), we evaluated the effect of WB-EMS with or without adjuvant easy leg movements in a supine lying position on maximum leg extensor and flexor strength. In summary, we observed a significant, twice as high, effect when WB-EMS was conducted during movements (i.e., active vs. passive mode). Of importance, movements *per se* had no effect on strength developments in this cohort of older women with sarcopenic obesity (Kemmler et al., 2015). This result confirmed the outcome of no to marginal effects on strength development of the adjuvant gentle, movements/exercises as applied in other studies (Kemmler et al., 2010c), even in less physically active or frail cohorts.

### General Considerations

This latter aspect leads us to an important methodological issue. In a recent systematic review Pano-Rodriguez et al. (2019) lament the general lack of comparability of WB-EMS and control groups in current studies that might confound the proper effects of isolated WB-EMS. Correspondingly, WB-EMS and control should conduct the same voluntary exercises ideally with and without WB-EMS switched on (1) to determine the net effect of WB-EMS but also to (2) blind participants by a placebo intervention. With respect to participant blinding, some authors (Jee, 2019; Kim and Jee, 2020; Ricci et al., 2020) have implemented corresponding placebo CGs; however, none of the studies reported whether blinding was successful. Taking the common sense of the participants into account, we feel that this procedure is easy to see through. Our blinding strategy within the TEST II and TEST III focused on strict separation and a control group with an attractive intervention, but with no or only marginal effect on body composition and functional outcomes. However, personal interviews revealed that most participants were aware that they had not been in the primary intervention group, thus we dispensed with this extensive approach in further studies (FORMOsA, FranSO). Summing up the issue of adequate control groups, we did not share the opinion of Pano-Rodriguez (Pano-Rodriguez et al., 2019) that the effect of WB-EMS was generally confounded,^10^ when the CG did not conduct the same, ultimately ineffective, voluntary movements as applied for functional aspects during WB-EMS. On the other hand, we do agree that a corresponding control group must be established when applying resistance or endurance exercise protocols (i.e., Amaro-Gahete et al., 2019a,b; Pano-Rodriguez et al., 2020a,b) superimposed by WB-EMS. However, we feel that superimposing already highly effective conventional exercises programs by WB-EMS does not fit the character and philosophy of WB-EMS as a perceived time-efficient, joint “friendly” option for people unmotivated or unable to exercise conventionally to increase their health and fitness status. Considering further that a large part of the training effect is generated by the voluntary workout, there might be little potential for further WB-EMS-induced effects (ceiling effect), an aspect that might relevantly confound the proper determination of WB-EMS effects on a given outcome.^11^

### Study Characteristics and Limitations

Apart from the exclusion of studies that superimpose intense exercise as the primary intervention by WB-EMS, there are several other features and limitations of the present study that should be addressed in order to allow the reader to comprehend our proceeding.

Considering that results of meta-analyses are significantly influenced by the studies included, it is a daunting task to select fully eligible studies particularly in the area of exercise interventions (Kemmler, 2013; Gentil et al., 2017). Although we placed high emphasis on including suitably comparable trials that represented the proper character of WB-EMS, with WB-EMS as the dominant agent, our inclusion strategy might have failed in some cases. This refers particularly to the studies of Bellia et al. (2020) and Ricci et al. (2020) with their specific co-interventions (bariatric surgery, energy restriction) on body composition. (3) We did not perform sub-analyses, e.g., in order to determine the most promising WB-EMS protocol (Gentil et al., 2017). Although the rather homogeneous impulse protocol of the included study might have allowed such an analysis, we conclude that the varying framework of the studies (see above) prevents a meaningful analysis. Further, in parallel to other types of exercise, it is unlikely that there is a “one size fits all” protocol for WB-EMS. This might be indicated by the dose-response study of Jee (Jee, 2019) that addressed impulse intensity with varying results even for related muscular parameters (Figure 2 vs. Figure 6). Further, apart from effectiveness, advanced safety aspects should be considered particularly when applying WB-EMS to older or even vulnerable cohorts. As an example, independent of potentially higher effectiveness, the application of (very) high impulse intensities (Jee, 2019) and/or frequent WB-EMS application (Kim and Jee, 2020; Ricci et al., 2020) conflicted with the safety aspects recommended by the German guideline (Kemmler et al., 2016a). (3) We do not share the opinion that resistance exercise and WB-EMS have to be considered as competing training methods, rather WB-EMS might be a time-effective and joint-friendly option to intensive resistance exercise. We do not aim to compare both methods in this context,^12^ nevertheless a rough comparison might be interesting for the reader. Confirming the data of a recent study (Kemmler et al., 2016b), absolute effects on muscle mass parameters (0.9 kg, 95% CI: 0.3–1.5 kg; Table 2) are in line with a recent meta-analysis on resistance exercise and LBM (1.1 kg, 95%-CI: 0.9–1.2 kg; Peterson et al., 2011) at least when considering that four out of 13 studies reported appendicular skeletal muscle mass changes. (4) Addressing the generalizability or external validity of our results, some restrictions also have to be stated. First of all, our data can only be referred to WB-EMS protocols with no, minor, or moderate relevance of the (gentle) voluntary exercises performed during the WB-EMS application. However, to our best knowledge, this is by far the most widespread WB-EMS strategy in the health and fitness domain. Apart from differences in age, a considerable number of studies included (11 of 18)^13^ focused on specific cohorts or conditions (i.e., sarcopenia, obesity, metabolic syndrome, tumor patients, back pain patients), which is an aspect that further challenges generalizability of our results. Nevertheless, we consider particularly older, less resilient, and physically limited cohorts with their low enthusiasm for exercise as one of the most important and challenging groups for WB-EMS application—at least from a health and socio-economic perspective. In conclusion, we provide further evidence for a significant positive effect of WB-EMS on muscle mass and muscle strength parameters, but not on total body fat mass in non-athletic adults. More dedicated studies should focus (a) on optimum WB-EMS protocols^14^ for given outcomes and varying target populations. Here the focus should be especially on WB-EMS application for: (a) diseases (e.g., multiple sclerosis, diabetes mellitus, selected types of cancer, hypertonia, arthritis) with limited potential or perspective for conventional exercise. (b) Intensity regulation by objective strain parameters that are based on advanced biomarkers to increase the safety and effectiveness of WB-EMS. (c) Long-term effects of WB-EMS with respect to safety and effectiveness. (d) Combination of WB-EMS with other low-threshold interventions (e.g., amino acid, creatine, ecdysteroid supplementation).

**Table 2:** Assessment of risk of bias for included studies (*n* = 18) according to PEDro and TESTEX scale.

**References**	**Eligibility criteria**	**Random allocation**	**Allocation concealment**	**Inter group homogeneity**	**Blinding subjects**	**Blinding personnel**	**Blinding assessors**	**participation≥ 85% allocation**	**Intention to treat analysis ^**1**^**	**Between group comparison**	**Measure of variability**	**Total score PEDro**	**Activity monitoring in control groups**	**Relative exercise intensity**	**Exercise volume and energy expended**	**Total score TESTEX**
Bellia et al. (2020)	Y	1	0	0	0	0	0	0	0	1	1	3	0	1	1	9
Jee (2019)	Y	1	0	1	1	0	0	1	0	1	1	6	1	1	1	10
Kemmler et al. (2018b)	Y	1	1	1	0	0	1	1	1	1	1	8	1	1	1	15
Kemmler et al. (2018a)	Y	1	1	1	0	0	1	0	1	1	1	7	1	1	1	14
Kemmler et al. (2016c)	Y	1	1	1	0	0	1	1	1	1	1	8	0	1	1	14
Kemmler et al. (2016d)	Y	1	1	1	0	0	1	1	1	1	1	8	0	1	1	14
Kemmler and von Stengel (2013) and Kemmler et al. (2015)	Y	1	1	1	1	0	1	0	1	1	1	8	0	1	1	13
Kemmler et al. (2014, 2015)	Y	1	1	1	1	0	1	0	0	1	1	7	0	1	1	12
Kemmler et al. (2010a, 2015)	Y	1	1	1	1	0	1	0	0	1	1	7	0	1	1	12
Kemmler et al. (2010b, 2015)	Y	1	0	0	0	0	1	1	1	1	1	6	0	1	1	12
Kim and Jee (2020)	Y	1	0	1	1	0	0	1	1	1	1	7	0	1	1	10
Ludwig et al. (2019)	Y	1	0	1	1	0	1	1	0	1	1	7	0	1	1	10
Ricci et al. (2020)	Y	1	1	1	1	0	1	0	0	1	1	7	0	1	1	12
Schink et al. (2018)	Y	0	0	1	0	0	0	0	0	1	1	3	0	1	1	9
Vatter (2010)	Y	0	0	1	0	0	0	0	0	1	1	3	0	1	1	7
Weissenfels et al. (2018)	Y	1	1	1	0	0	1	1	1	1	1	8	1	1	1	15

*Y, yes*.

## Conclusion

Although this systematic review and meta-analysis provided further evidence of WB-EMS effects on body composition and strength, its generability refers predominately to moderately old to older untrained or at least non-athletic cohorts. Further, one should consider that the present results were only attributable to WB-EMS protocols that focus on moderate to high impulse intensity and low to negligible voluntary workload, an approach to our best knowledge used by the vast majority of commercial and clinical WB-EMS settings, however. Deviating from our results, a previous (mini) meta-analysis (Wirtz et al., 2019) that addressed superimposed WB-EMS with high intensity voluntary workload protocols in athletic cohorts, resulted in non-significant WB-EMS effects. This result might relate to the problem of detecting small but nevertheless important changes in performance parameters in athletic cohorts with their limited potential for further improvements. Thus, a dedicated comprehensive meta-analysis that generates sufficient statistical power should address the effects of superimposed WB-EMS in athletic cohorts to conclude this issue.

## Data Availability Statement

The raw data supporting the conclusions of this article will be made available by the authors, without undue reservation.

## Author Contributions

WK, MS, JS, JB, MF, DS, SS, HK, and MK completed data analysis, interpretation, and drafted the manuscript. All the authors contributed to study conception and design and revised the manuscript. WK accepted responsibility for the integrity of the data sampling, analysis, and interpretation.

## Conflict of Interest

The authors declare that the research was conducted in the absence of any commercial or financial relationships that could be construed as a potential conflict of interest.

## Footnotes

^1^…. below muscular adaptation threshold.

^2^At least when applying the more popular concept with low to negligible workloads.

^3^This included impulse parameters (type, breadth, frequency, increase, load cycle), length and frequency of WB-EMS application, and general exercise intensity. See also Table 1.

^4^i.e., voluntary loads, or more precise movements that *per se* should not or at least not relevantly affect primary study outcomes.

^5^However, it is not clear whether WB-EMS was applied for the entire 40 min exercise with music session.

^6^Impulse intensity was gradually increased up to an unbearable level (100% maximum tolerance).

^7^However, a separate analysis that did not include the studies of Bellia et al. (2020), Ricci et al. (2020), and Schink et al. (2018) also resulted in non-significant results (-0.59; 95% CI: 0.17 to −1.36) and substantial heterogeneity between the trials (Q: 51.7; *I*^2^: 89.6%).

^8^It remains unclear whether WB-EMS was applied during the entire 40 min session.

^9^In contrast to Bellia et al. (2020), Willert et al. (2019) further provided whey protein supplements for all study groups and generated negative energy balance differently for the CG (energy restriction) and the WB-EMS group (energy restriction and physical activity). Due to this complex protocol we decided against including this study in the analysis.

^10^At least on body composition and functional outcomes.

^11^This aspect ultimately leads to the exclusion of studies with conventional exercise as the primary intervention.

^12^..this should be done by more dedicated clinical studies (e.g., Kemmler et al., 2016b).

^13^At least when considering obesity still as a “specific condition.”

^14^This particularly refers to impulse parameters including wave forms, but also to the volume and general intensity of the WB-EMS application.
